# Supplementary material for: Optimization of a Three-Dimensional Culturing Method for Assessing the Impact of Cisplatin on Notch Signaling in Head and Neck Squamous Cell Carcinoma (HNSCC)
Source: Cancers (Basel). 2023 Nov 7;15(22):5320. doi: 10.3390/cancers15225320 (PMC10670464; doi:10.3390/cancers15225320)
Supplement: Supplementary file 1 [file cancers-15-05320-s001.zip › Anameric et al 2023 Supplemental Figures.pdf]

Supplemental Figures

Article

Optimization of 3D Culturing Method for Assessing Cisplatin's Impact on Notch Signaling in Head and Neck Squamous Cell Carcinoma (HNSCC)

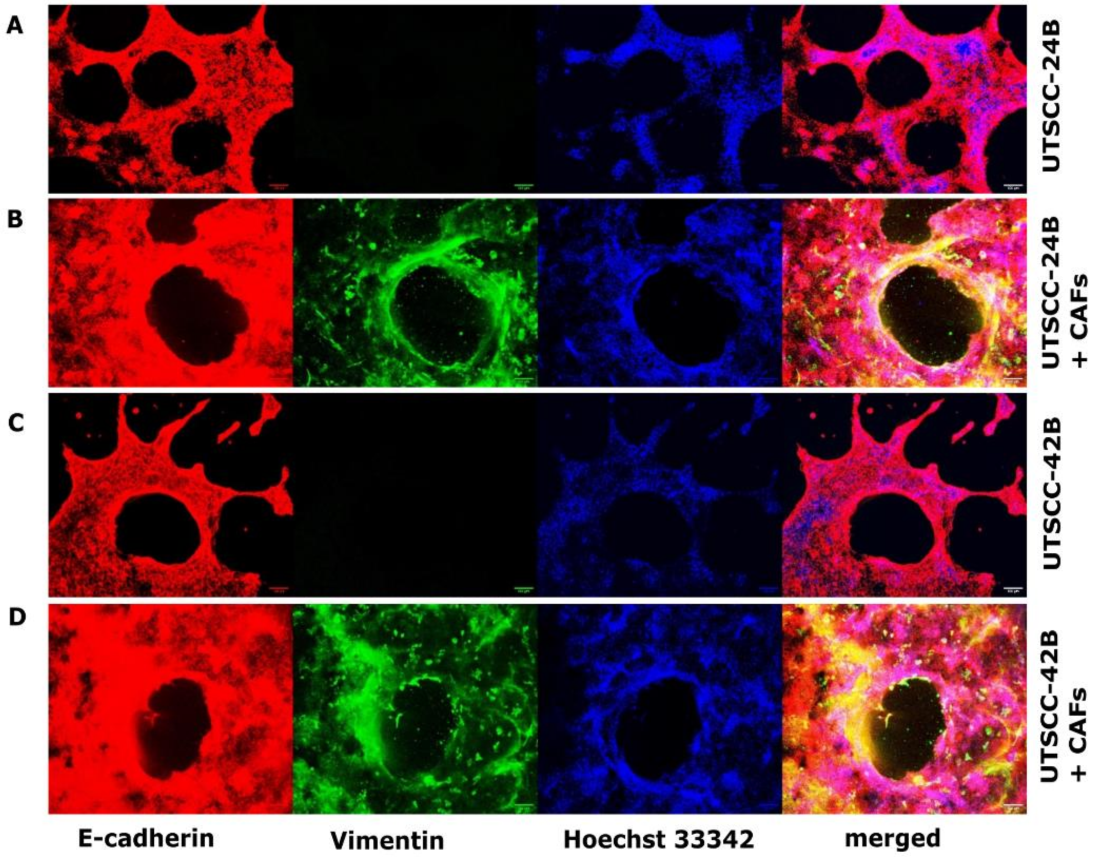

Supplemental Figure S1: Analysis of multicellular, tissue-like aggregates forming in 3D co-culture of tumor cells (UTSCC-24B and 42B) with patient-derived CAFs in the "3D sheet model". A) 3D Monoculture of UTSCC-24B tumor cells, B) Cocultures of UTSCC-24B tumor cells and patient-derived fibroblasts (1:1 ratio) C) Monocultures of UTSCC-42B tumor cells, D) Cocultures of UTSCC-42B tumor cells and patient-derived fibroblasts (1:1 ratio). All structures form on the surface of a Matrigel/collagen type I gel. The structures resemble the cribriform morphology observed in different epithelial tumors, including HNSCC. Red color represents E-cadherin, green color represents vimentin and blue color represents Hoechst 33342. Confocal images were taken on day 7 after seeding at 10x magnification with a NIKON confocal microscope.

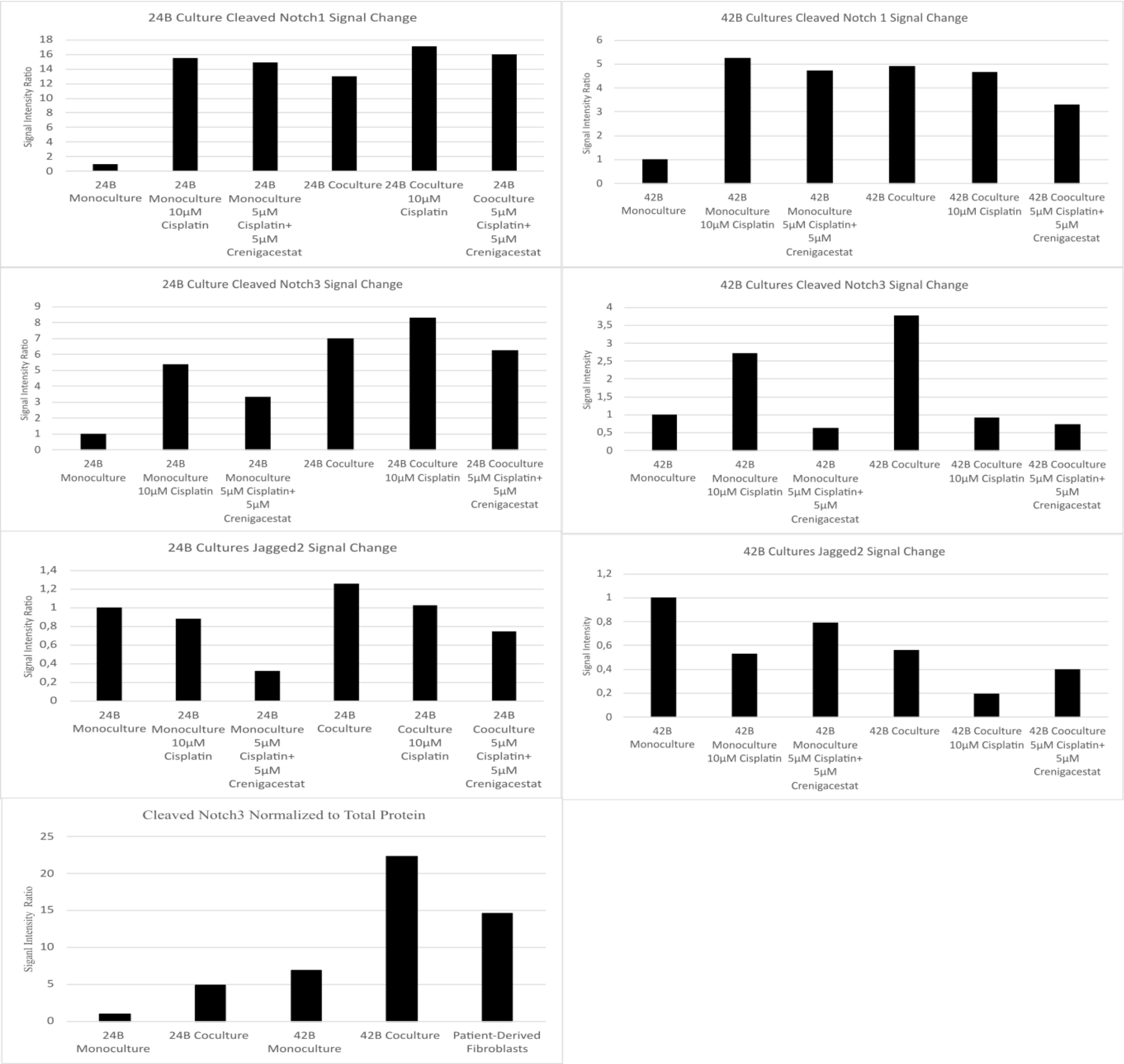

Supplemental Figure S2: Densitometry analyses of the Western blot results shown in Figure 7. Detailed description in Fig. 7 of the main article.
